# Public Transportation Environment and Medical Choice for Chronic Disease: A Case Study of Gaoyou, China

**DOI:** 10.3390/ijerph16091612

**Published:** 2019-05-08

**Authors:** Yang Cao, Feng Zhen, Hao Wu

**Affiliations:** 1 School of Architecture and Urban Planning, Nanjing University, Nanjing 210023, China; libracaoyang@163.com; 2 School of Atmospheric Sciences, Nanjing University, Nanjing 210023, China; dione.wu@foxmail.com

**Keywords:** chronic disease, medical choices, built environment, driving forces, Gaoyou

## Abstract

Current research on the built environment and medical choice focuses mainly on the construction and optimization of medical service systems from the perspective of supply. There is a lack of in-depth research on medical choice from the perspective of patient demand. Based on the medical choice behaviour of patients with chronic diseases, this article identifies the spatial distribution and heterogeneity characteristics of medical choice and evaluates the balance between medical supply and demand in each block. On this basis, we explored the mechanism of patient preferences for different levels of medical facilities by considering the patient’s socioeconomic background, medical resource evaluation, and other built environment features of the neighbourhood by referring to patient questionnaires. In addition to socioeconomic characteristics, the results show that public transportation convenience, medical accessibility, and medical institution conditions also have significant influences on patient preferences, and the impact on low-income patients is more remarkable. The conclusions of the study provide a reference for the promotion and optimization of the functions of urban medical resources and the guidance of relevant public health policies.

## 1. Introduction

Medical treatment behaviour mainly refers to the behaviour of seeking medical help when people feel unwell or have symptoms of certain diseases. It is an individual’s choice of behaviour in the context of the public health care system and also taking into account the patients’ own socio-economic background and living environment. It is a process involving patient behaviour and psychological integration [1]. The main related research topics include the timing of patient treatment, the choice of medical institution, the choice of medical staff and the choice of drugs [2]. International studies on medical treatment behaviour are abundant. There are four types of influencing factors that affect individual medical treatment behaviour, including family demographic characteristics, economic factors, medical quality and patients’ medical needs. The study found that family demographic characteristics, including age, gender, education level, marital status, economic constraint etc. are significant factors affecting individual medical behaviour [3,4]. The time cost of medical services, including transportation time and waiting time for medical treatment also affect individual medical decision-making [5,6]. Many studies have also found that the quality of service, medical charge and other factors of medical institutions also have an impact on patients’ choice of medical treatment [7,8,9]. Studies have also shown that the health conditions, self-evaluation disease status and other medical needs also affected the medical choices [10,11].

Among these stages, the choice of medical institution (i.e., medical choice) reflects patients’ comprehensive understanding of their own diseases and the medical facilities, as well as the impact of the medical facilities’ layout on patient behaviour. With respect to medical choice, existing research has focused on two aspects. The first is the impact of medical infrastructure conditions on a patient’s medical treatment behaviour considering the medical institutions’ qualifications, costs, environment, and accessibility [6,12,13]. The other is the differences in institutional choice preferences among different types of patient groups, starting from the patient’s socioeconomic background and cultural concepts [10,11,14,15]. The existing research on medical choice focuses on sociology, economics, education and other fields. There is relatively little research on the relationship between medical choice and built environment features in the field of health geography. The combination of specific policy application levels, such as the planning and construction of medical facilities, is insufficient. In terms of mechanism analysis, the existing research also lacks comprehensive consideration of subjective and objective factors, such as traffic convenience, facility quality, living environment, family background, and daily patient behaviours [5,16].

Chronic disease is a general term for chronic non-communicable diseases. According to the definition provided by the World Health Organization, chronic diseases mainly refer to a group of diseases represented by cardiovascular and cerebrovascular diseases, tumour diseases and chronic organic diseases caused by factors, such as environmental hazards and lifestyle [17]. This article focuses mainly on the following chronic diseases: Diabetes, hypertension, and hyperlipidaemia. The chronic disease population is concentrated in the middle-aged and older populations, and the disease state usually lasts for a long time [18]. The aetiology of chronic diseases is characterized by complexity, and there are often no specific sources or causes of infection. Studies have shown that chronic diseases are associated with the patient’s living environment, dietary habits, and family genetic factors. Compared to other patients, those with chronic diseases have more demand for medical institutions and need to seek medical care over a longer period of time. According to the requirements of the Chinese government’s medical and health policy, the government encourages patients with chronic diseases to choose hospitals around the residential area for medical treatment. The primary care institutions on community-level are required to meet more medical needs of patients, thereby alleviating crowding and resource constraints in large medical institutions. Moreover, the aetiology of chronic diseases is complex. In addition to family genetics, dietary habits and other factors, chronic diseases are also influenced by medical choice, medical services, and medical review frequency [19].

Research on the built environment and medical choice focuses mainly on medical resource allocation, medical service supply levels, and medical service accessibility. Studies tend to focus on influencing factors, such as the coverage of medical facilities, road connectivity, and the total population of the district from the supply perspective [20,21,22]. Generally, geospatial analysis and other methods are used to measure the accessibility and balance of facility layouts and assist in the formulation of public policies related to issues, such as facility layout. However, the existing facility evaluation research from the demand perspective is limited by the available data acquisition tools, such as statistical yearbooks and questionnaires. The accuracy and timeliness of the data are relatively insufficient, and the attribute dimensions are not rich enough. It is difficult to describe the specific use process of a facility [23,24]. At present, nearly all levels of medical and health institutions in China keep complete patient medical records using information platforms. By correlating the socioeconomic attributes of patients, it is possible to study the specific influencing factors of patients’ medical choice from an individual perspective. This approach provides a new method for the spatial layout and functional optimization of medical facilities from the perspective of individual patient needs.

**Figure 1:**
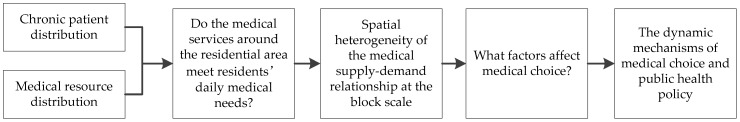
Overall research framework.

This paper takes Gaoyou City in Jiangsu Province as the study area and identifies the spatial distribution characteristics of chronic disease-related medical choice. Based on medical choice behaviours related to chronic diseases, this article identifies the spatial characteristics of medical choice and evaluates the balance between medical and living space in each block. On this basis, in view of the spatial heterogeneity characteristics of medical service supply and demand, we explore the mechanism of patient preferences for different levels of medical facilities considering the patient’s socioeconomic background, medical resources, and the built environment of the neighbourhood with reference to the patients’ questionnaires (Figure 1). The rest of the study is organized as follows. Section 2 describes the study area, the sources and processing of the data and the statistical analysis method. Section 3 illustrates the spatial characteristics of and factors driving medical choice for chronic diseases. Section 4 discusses the influencing factors and mechanisms of chronic disease medical choice and provides policy implications. Section 5 draws conclusions based on the results and discussion.

## 2. Methods and data

### 2.1. Study Area

**Figure 2:**
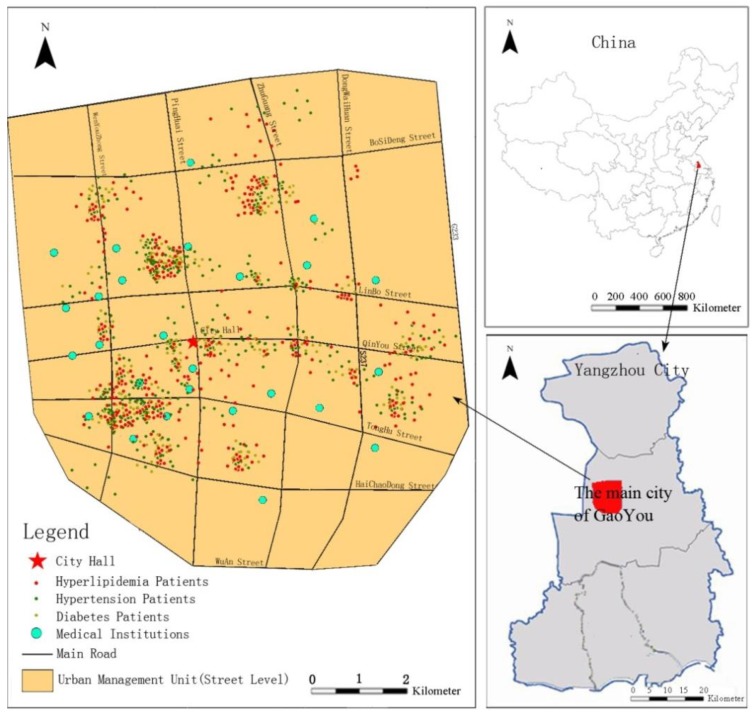
Location of Gaoyou and the study area.

The main urban area of Gaoyou City (under the jurisdiction of Yangzhou City) in Jiangsu Province was chosen as the case study area. This city has a population of approximately 330,000 and a total area of 78.12 km^2^ (Figure 2). With the rapid development of the local social economy in recent decades, considerable changes have taken place in the urban structure and socioeconomic environment, such as rapid population agglomeration in central urban areas, and many large-scale restructuring and renovation projects have been carried out in the built environment at the block scale. The existing urban medical resources were distributed in blocks to address the dual needs of functional upgrades and layout optimization. We selected 34 blocks within the main city of Gaoyou as the smallest research unit and specifically analysed the characteristics of medical choice and the influencing factors with relation to chronic diseases in each block. This study chooses the city block as the research unit, mainly refers to the area surrounded by four streets and the shapes are usually square or rectangular. The block can also be delimited by other visible or invisible natural or human characteristics, such as administrative boundaries, rivers, lakes, railways, mountains, cliffs, etc. In addition, the block unit is normally used as a basic unit to clarify the status and planned land type and statistics on the size of the population in urban planning. The government also allocates corresponding public service resources in the block, such as transportation, medical care, education, etc.

### 2.2. Data Collection

The research data in this paper include mainly patient attribute data, medical record data, medical institution evaluation data and the built environment data for the study area. Specifically, (1) we chose chronic diseases (hypertension, diabetes and hyperlipidaemia) as the research subjects. We obtained the 2016 city-wide patient medical outpatient records from the municipal health department. The records cover information, such as the type of illness and the choice of medical institutions. Due to the presence of multiple chronic diseases in individual patients, we ultimately obtained a total of 2356 records of patients with chronic diseases, and a total of 19,568 medical records were collected for all of 2016. Our research objects are the whole patients of chronic diseases. We didn’t distinguish the patients according to the type of disease like diabetes, hypertension and hyperlipidaemias. After we identified 2356 patients with chronic diseases through medical records, we distributed questionnaires to this group to collect the information of the patients’ socio-economic background, evaluation of medical institutions and the traffic environment around the residential areas, etc. The number of 19,568 refers to the patients’ medical records, not medical documents. Since it forms a medical record during each medical treatment each, the number of medical records will be greater than the number of patients. We have recovered 1485 valid questionnaires and screening out the samples using public transportation for medical treatment. Finally, the sample size that eventually entered the regression model was 1139, which accounts for 76.7% of the valid questionnaires. (2) We also conducted a questionnaire based on the residential information in these patients’ medical records to obtain individual patient attribute information, self-rated disease severity data, and subjective medical facility perception scores. In 2016, the research team conducted a questionnaire on the patients’ medical behaviour with chronic diseases (see Appendix A). The investigation time is from February to May 2016. A total of 1741 questionnaires were distributed and recovered 1485 valid questionnaires actually. The effective recovery rate of the questionnaire was 85.3%. We have carried out corresponding work in the questionnaire design stage, in the questionnaire survey stage, and the data processing stage of the questionnaire. Specifically, in the questionnaire design stage, the questionnaire conducted several rounds of discussions within the research team and sought the opinions of medical psychology and medical ethics experts. Ethics approval was obtained from the Medical Ethics Committee of Gaoyou Health Bureau. At the beginning of the symposium, the medical ethics experts were provided with a written research content schedule and a verbal explanation of the research purpose. Then we received the ethical committee’s approval. This study drew on the research specification of the existing achievements [25,26] and the study was conducted in accordance with the 1995 Helsinki Declaration [25]. In the questionnaire survey stage, we distributed 100 questionnaires for pre-study and all subjects gave their informed consent for inclusion before they participated in the study to ensure that the content of the questionnaire can be accepted by patients. The specific instructions are also included in the text of the questionnaire that “we confirm that the results will be only used for academic statistical research rather than other use in any form. Your participation is anonymous. If you have any discomfort during the questionnaire process, you can interrupt it at any time. We promise not to report or disclose your personal information in any way.” Participants gave consent to participate in the study on the survey of individual socioeconomic background, medical resource evaluation and built environment assessment. Notably, to protect the privacy of patients, the patients’ name attributes were actively removed during the data processing. This paper mainly studies the common characteristics of patients’ medical choices with similar socio-economic background and built environment conditions. (3) In addition, a total of 45 medical institutions at different levels within the study area were selected; their spatial location and basic attribute information were collected through the Internet, and their space and attribute information were mapped using a GIS platform. Notably, to protect the privacy of patients, the patient name attribute was actively removed when acquiring the data. The study solely explores the characteristics and mechanisms of group activities and does not elaborate on individual patients. In addition, we mainly used medical records to identify patients with chronic diseases. The information about the patients’ own attributes, subjective evaluation of medical institutions and the built environments around the residential area are obtained through the questionnaires. We asked the interviewees to complete the questionnaire as completely as possible. We also linked the patients’ medical records with their questionnaire survey data through the patient’s ID number. We analysed the medical behaviours of patient groups with similar attributes by the regression model. Group activities refer to patient groups with similar socioeconomic backgrounds, medical service evaluations, or public transportation environments in residential areas.

Patients with high income levels often have a better ability to pay, and they may be more likely to choose formal medical services when disease symptoms appear. This paper uses the World Bank’s “$2/day” income poverty line to divide the sampled patients into poverty and non-poverty groups [27]. According to the 2016 purchasing power standard in China, the cut-off is 2854.3 yuan/year. Since chronic disease groups include mostly middle-aged and older persons, they depend primarily on walking and bus travel. Therefore, in the medical accessibility analysis, public transportation is selected as the common travel mode. In addition, since a subway has not been constructed, the study considers the average travel speed of urban vehicles based on the average speed of urban vehicles and estimates the travel time required for daily medical trips. The built environment conditions mainly measure the travel time of patients for medical treatment and evaluate whether they can obtain medical services faster by using public transportation. Specifically, the coordinates of the medical facility, the general bus travel time of the patient, and the walking time of the patient are obtained using an online map. The Online API refers to the application interface of the online map on the internet. China’s Baidu map (like Google map) provides a third-party application interface that allows users to call by writing programs. This study obtained the spatial location information of medical facilities, patient residences, road traffic, and public transportation facilities to measure medical accessibility through Baidu map Online API.

### 2.3. Variables to Describe the Potential Driving Forces

This article considers that medical choice factors are divided mainly into two categories: (1) Individual characteristics, including the patient’s age, sex, income level, health concept, type and severity of illness, medical insurance, and medical payment category [28]; and (2) the objective conditions of the medical institutions, including price, service quality, environment, location distance and traffic accessibility [29]. Existing research has overemphasized the influence of individual socioeconomic characteristics or other single indicators, such as the medical facility or medical accessibility, and lacks an overall consideration of the cross-effect relationship of multidimensional factors [30,31,32]. In addition, the construction of the corresponding models overlooks the interaction between mediator variables and regulatory variables, and these two variables have important implications for the synergistic effects of cognitive factors.

The type of medical institution was chosen as a dependent variable and divided into four categories: County-level hospitals and above, township-level hospitals, private clinics, and village-level hospitals. The village clinic is the lowest level of formal medical and health institution, operating under the village-level administrative system, and it is used as an experimental control group in the model. The model is divided into three categories of independent variables: Patient socioeconomic characteristic variables, medical institution characteristic variables, and built environment variables. (1) The socioeconomic factors of the patient specifically relate to the individual characteristics of the patient (age, sex, cultural level, and personal attributes, such as marital status) and patients’ self-rated health status (related mainly to the type and severity of the disease and participation in medical insurance) and economic background referring mainly to the average income level of the patient’s family (Table 1). (2) Medical institution factors include institutional fee self-evaluation, service self-evaluation, and environmental self-evaluation. From the perspective of patients’ use satisfaction with medical institutions, subjective satisfaction levels in the use of medical resources are investigated and divided into two categories: Satisfaction and dissatisfaction. (3) In addition to subjective factors, such as the patient’s economic level, health cognition level, and daily life habits, medical choice is also affected by the environmental factors of the surrounding entities in the neighbourhood. This paper specifically measured public transportation convenience and medical accessibility around the residential area (Table 2).

### 2.4. Data Analysis

**Figure 3:**
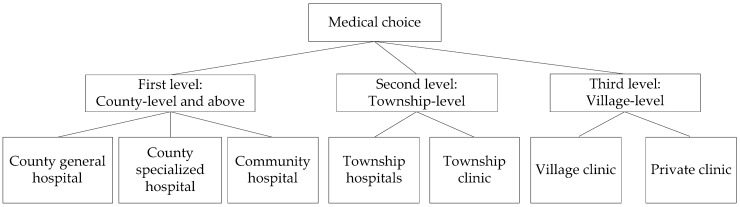
Nested decision tree for chronic disease medical choice.

The independent variables in this study include multidimensional variables, such as individual characteristic variables and institutional characteristic variables. If we selected the traditional multiple logit model, there would be problems, such as the assumption that the hypothesis test is too strict and the fit is not good. Considering the hierarchical relationship between medical institutions (Figure 3), this paper uses the conditional multiple logit model, or nested multiple logit model, to calculate the probability of individual medical choices [28,29]. The model assumes that the individual must determine the level to which a medical institution belongs before selecting the medical institution and then identify a specific medical institution at the level. When the random part of the utility function obeyed the Weibull distribution and was relatively independent, multiple logit models were selected as the control group (Equation (1)). The following formula was used to calculate the probability *P_ij_* that an individual will choose a different clinic; *i* refers to the individual sample, *j* refers to types of medical institutions, and *n* refers to individual feature attributes:

(1) $$P_{ij}=\frac{e^{{x_{i}}^{\prime }\beta_{j}}}{\sum_{m=1}^{n}e^{{x_{i}}^{\prime }\beta_{j}}},j=1,\ldots ,n.$$

Nested multiple logit models relax the IIA hypothesis. The types of medical institutions that may be related are classified as one category, and the relevant cases remain in the classified results to continue the iteration until there is no correlation between the categories, thereby solving the correlation problems between different options. The probability that the individual selects option *k* at level *j* can be expressed as follows:

(2) $$P_{jk}=P_{j}\times P_{k|j}=\frac{\exp(z_{j}^{\prime }a+p_{j}I_{j})}{\sum_{m=1}^{j}(z_{m}^{\prime }a+p_{m}I_{m})}\times \frac{\exp(x_{jk}^{\prime }\beta_{j}/p_{j})}{\sum_{l=1}^{K_{j}}\exp(x_{jl}^{\prime }\beta_{j}/p_{j})}.$$

*P_j_* represents the degree of correlation between nested internal options; when it is equal to 1, the options inside the nest are completely unrelated. This paper conducts empirical tests on models based on questionnaire data. Because the multi-logit model showed the greatest contrast, the interpretation of the three types of mathematical models was compared using goodness-of-fit indicators (Table 3). The larger the log-likelihood value, the better the goodness of fit. The smaller the values of the Akaike information criterion (AIC) and the Bayesian information criterion (BIC), adjusted according to the number of parameters and the number of samples, the better the model fit [32,33]. The goodness-of-fit indicators show that the multi-logit model has the poorest fit. The log-likelihood, AIC, and BIC values all support that the nested multi-logit model has the best fit. Thus, the nested model is the most reliable. Therefore, we chose the nested multiple logit model for the statistical analysis of the influencing factors.

This article explores the following questions related to using nested multi-layer models. (1) Do different social and economic backgrounds affect patients with chronic diseases who choose not to go to medical institutions, such as graded medical institutions or private clinics? (2) What socioeconomic attributes of patients and their families significantly affect their medical choices? How are they affected? (3) What built environment factors influence a patient’s choice regarding medical institutions? Do individual-level explanatory variables and a patient’s choice of medical care change with the characteristics of the residential environment and the surrounding environment of the medical facility? (4) Does poverty level cross-influence a patient’s medical choice with indicators, such as perceived medical service level and perceived health level?

## 3. Results

### 3.1. Spatial Distribution Characteristics of Medical Supply and Demand

**Figure 4:**
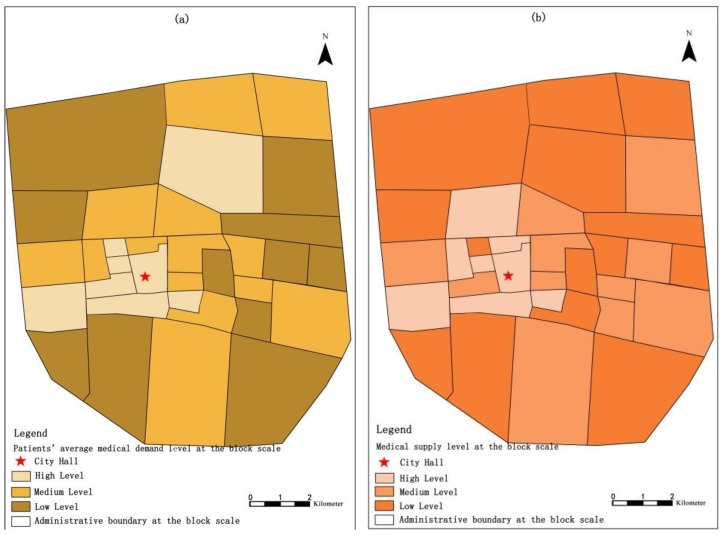
Residential agglomeration level (**a**) and medical supply level (**b**) in block units.

We take the block as a spatial measurement unit and calculate the distribution of patients’ average medical demand intensity based on their medical choices (Figure 4a), and we use the superimposed comprehensive value of each medical institution service level as the medical supply intensity in each block unit (Figure 4b). We unified the quantification of three levels, i.e., high, medium, and low, to facilitate the supply-demand balance comparison of medical choice at the block scale. According to Chinese government policy documents, the distribution of medical and health institutions at all levels in cities and towns should meet the medical needs of the residents in the districts under their jurisdiction as much as possible based on their service population and radius [21]. In this paper, descriptive statistical analyses were made on the 45 medical institutions covering each block in the study area (Table 4). The results show that (1) the blocks’ medical resource coverage level surrounding Gaoyou’s main urban area is relatively good, and these blocks have multi-level medical resource superimposed services. The supply of medical resources in the peripheral low-density residential areas is relatively insufficient. There is still no coverage of medical resources in individual neighbourhoods. (2) There is obvious spatial agglomeration of medical resource distribution, and this agglomeration first increases and then decreases with distance from the city hall. (3) Moreover, there are also large differences in the direction of spatial distribution.

### 3.2. The Balance of Medical Supply and Demand at the Block Scale

Considering that most patients with chronic diseases are middle-aged or older persons, the range of medical-related trips is limited, and chronic diseases primarily require high-frequency behaviours, such as returning to the clinic and taking medicine [33,34]. The analysis of the relationship between the supply and demand for medical and residential spaces at the district scale can more accurately reveal the current status of medical supply services. Starting from the analysis of the characteristics of medical institutions for chronic diseases, we measured the spatial balance of medical supply and demand in the main urban area at the block scale. This article constructs a medical supply and demand relationship classification table to analyse the medically balanced relationships at the block level. Medical resource supply and patient choice demand for medical treatment are divided into three levels (low, medium and high) and the differences between the supply and demand levels are compared. Obviously, when supply and demand belong to the same level, they are most likely to achieve a balance. The larger the difference in the level, the greater the imbalance. In theory, the supply and demand relationship can be divided into nine states (Table 5).

**Figure 5:**
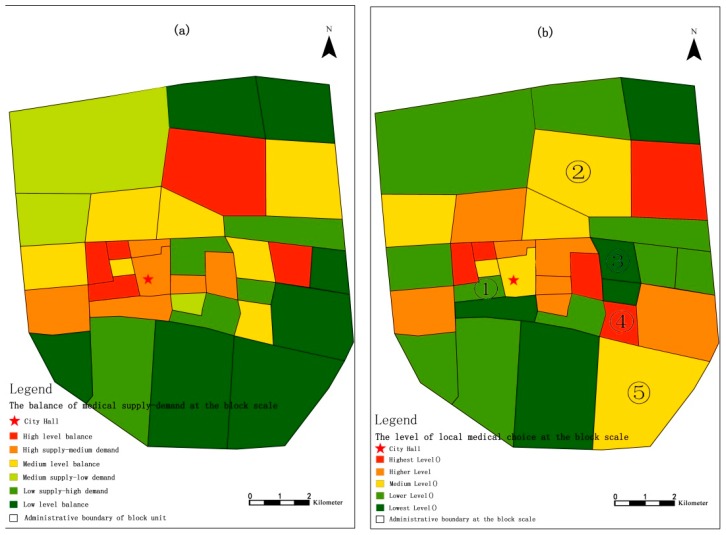
The balance degree of medical supply-demand (**a**) and local medical choice level (**b**).

We pay special attention to the comparison of patients’ behaviours in medical institutions in their neighbourhoods and non-residential medical institutions. We separated the balance degree of medical supply-demand (Figure 5a) and local medical treatment level (Figure 5b) in the block unit. The analysis revealed the following. (1) There are 16 blocks in the main urban area that exhibit medical supply and demand balance at different levels, but there are 18 other blocks showing a mismatch in the medical supply-demand relationship. Among them, there are four blocks with significant medical supply and demand imbalances of “low supply—high demand”. (2) Moving outward from the city centre, the medical supply and demand balance is generally low. This result reflects the lack of allocation of medical resources in the residential community, and patients are more willing to choose large-scale general hospitals with complete functional facilities in urban areas.

In addition, (3) there is an imbalance between the actual use level and supply level of medical resources within each block. Although the medical resource allocation intensity of blocks ① and ② in the figure is high, the medical treatment rate of local patients is not high. The allocation of medical resources in blocks ③, ④, and ⑤ is not high, but the local medical treatment rate is relatively high. To a certain extent, these findings reflect that the medical services of these clinics basically meet the needs of local patients, allowing the corresponding neighbourhoods to maintain a good medical and housing balance. To further analyse the causes of the medical and housing imbalance in some neighbourhoods, we explored the associations of socioeconomic background, type of illness, quality of hospital services and the built environment. Based on the results of the feature analysis, we further explored the medical choices of patients exhibiting an imbalance in the neighbourhoods and explored the specific influencing factors.

### 3.3. Driving Forces of Medical Choice Related to Chronic Illnesses in Gaoyou City

The descriptive statistical results indicate that males in the sample are more likely to choose medical institutions at or above the county level than female patients. Patients over the age of 60 tend to choose small medical institutions, such as township hospitals. Patients with a high school education and above are more likely to choose hospitals at or above the county level. Patients with high income tend to choose a higher level of health services. The model results revealed that socioeconomic background factors, medical institution evaluation factors and various built environment features had different degrees of influence on medical treatment choices (Table 6). In terms of individual socioeconomic background, regarding patient socioeconomic attributes, the model results reflect that patients’ age, sex, education level, income level, self-rated disease severity and poverty have a significant impact on their choice of medical institution. In addition, medical insurance involves the form that patients’ medical expenses take and the proportion of medical expenses reimbursed, playing an important role in patients’ choice of medical institution. In terms of built environment features, medical institution evaluation, public transportation convenience and medical accessibility have varying degrees of influence on patients’ medical choices.

## 4. Discussion

### 4.1. The Relationship between Individual Characteristics and Medical Choices in Gaoyou

We found that a young age (31–45 years old) has a significantly positive impact on the decision to visit a township hospital. This finding shows that younger adults are more inclined to choose a nearby township hospital and other residential areas than visit the village clinic. The middle-aged group (45–60 years old) has a negative coefficient for private hospitals, while the coefficient for county-level hospitals is positive and statistically significant. This finding shows that patients visit formal medical institutions, such as village clinics more frequently than private clinics and are more willing to go to a high-level institution. Compared with female patients, males have a significantly higher probability of choosing a medical institution at or above the county level. This finding is consistent with the statistical results of the sample description and with the existing research results [35,36]. Patients with low education levels (primary and junior high school) have a higher probability of choosing private clinics, and patients with higher education (high school and above) are more likely to choose county-level hospitals. Patients with lower household incomes (below 5000) have a higher probability of choosing private clinics, while those with higher incomes (more than 5000) have a higher probability of choosing hospitals at the county level; these results are statistically significant. This outcome is inconsistent with international research conclusions about patients of the same types [37], primarily because of the differences in national conditions between China and Western countries. Western countries adopt market-oriented modes of operation in public service industries, such as healthcare and provide differentiated medical service choices for different groups. China has experienced rapid development in the past 20 years, and the country’s annual growth rate of GDP is between 5% and 7%. Rapid urban development has increased the income of residents, but it has also created other social problems, such as expanding the polarization between rich and poor. In addition, due to the different national political systems, China’s health care system policies are quite different from those of Western countries. At this stage, China has a large population, but the overall medical security system of the residents is imperfect and the distribution of medical resources is uneven. The state focuses on the development of large public medical institutions to meet the basic medical needs of a wider population which has led to a higher level of medical conditions in public hospitals. However, private hospitals receive weaker government support in terms of funds, personnel, policies, etc., and the medical conditions of the institutions are correspondingly poor. It is difficult for low-income patients to afford higher medical charges, so they mainly choose private medical institutions with lower fees. The high-income people are more willing to choose a large public medical institution with better conditions”. In addition, we gave the further explanation in the limitation section that: “This study mainly used the one-year cross-sectional data to reveal the diverse factors influencing patients’ medical choice. In the next study stage, we will continue to research the patients’ medical behaviour for many years and try to reveal the mechanism of patients’ income changes to their medical choices. We believe that it may be an interesting study. The results of the model also show that patients with medical insurance have a significantly higher probability of selecting high-level medical institutions, showing that insurance plays a prominent role in promoting the choice of high-level medical institutions. In terms of self-reported disease severity, patients with less severe diseases have a significantly higher probability of choosing township hospitals and private institutions. When the disease is serious, patients are more inclined to choose high-ranking hospitals at or above the county level, showing an obvious statistical correlation. We incorporated the length of time since diagnosis into the analytical model. The results show that the patients who were diagnosed with less than one year to live were more likely to choose township-level hospitals and county-level hospitals. The patients who were diagnosed more than two years to life were more likely to choose private clinics for medical treatment.

### 4.2. The Relationship between the Built Environment and Medical Choices in Gaoyou

At the level of medical resource attributes, dissatisfied patients are significantly more likely to choose higher-level medical institutions at or above the county level. In the case of patients who are satisfied with the level of medical services, township hospitals and private clinics are significantly more likely to be chosen. This reflects the impact of medical costs on the high-level medical treatment choices of patients. Medical outcomes and service quality showed significant correlations among different types of medical institutions, and the cost of medical services especially affected the poor income groups. In addition, the cross-term factor analysis results show that poor families are significantly more likely to choose medical facilities at or above the county level. In the case of satisfactory medical services, the choice of private clinics is more probable and statistically significant. With respect to build environment factors, the convenience of residential buses and the medical accessibility index of residents in residential areas are significantly correlated with patients’ choices. This conclusion is consistent with the existing research on the medical service needs of outpatients in rural areas [38,39,40]. In the case of a bus travel time greater than 10 min, the probability of patients choosing a medical institution above the county level is higher and statistically significant. In addition, the coefficient for choosing a private clinic is positive for bus trips under 10 min and negative for those longer than 10 min. This finding shows that, in the case of increased travel time, patients are more inclined to choose a regular medical institution (village clinic) and exhibit statistically significant features at the 15-min level. For medical accessibility indicated by a bus ride less than 10 min, the probability of patients choosing private clinics and county hospitals is significantly higher. In the case of a ride longer than 10 min, the patients were compared to those choosing private clinics and preferred to choose a village hospital for medical treatment, and the statistical characteristics were significant when the bus ride was 15 min or longer. This pattern shows that patients have a statistically higher probability of choosing medical institutions at or above the county level than village clinics. It also supports the characteristics of the influence of bus convenience on the choice of medical institutions [41].

### 4.3. The Mechanisms Influencing Medical Choices for Chronic Diseases in Gaoyou

**Figure 6:**
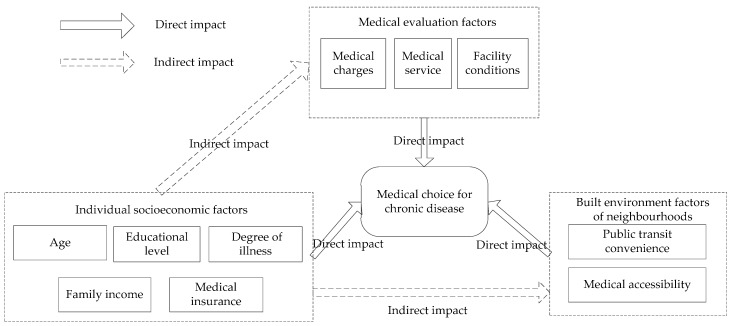
The mechanisms influencing medical choices for chronic diseases in Gaoyou.

The above analysis shows that patients’ choices regarding medical treatment are influenced by individual socioeconomic attributes, medical and health resources, and the environmental features of residential areas. There are also interactions between individual factors, medical resources, and the environmental features of residential areas. (1) First, the individual socioeconomic attributes show strong coupling with the convenience of public transportation and medical convenience indicators in the residential area. Therefore, patients’ choice of medical institution varies under different built environment conditions. (2) Second, the current level of coverage of China’s medical insurance system and the differences in the level of reimbursement across different types of medical insurance are gradually narrowing. However, patients of different socioeconomic backgrounds also have significant differences in cognitive level and the ability to choose medical facilities. Medical institutions at different levels have relatively large differences in the level of medical treatment quality, medical charge, and the environment of the institution itself. The original medical institution level still has a restraining effect on the medical choice process of patients. (3) Patients in a similar socioeconomic context tend to choose the same type of medical institution, while the same type of residential environment leads to having similar surrounding medical institutions (Figure 6). Especially in the built environment, the medical resource level and the built environment features of neighbourhoods explain the background effect of the differences in individual medical treatment behaviour to some extent, which means that patients with similar living conditions are more convergent in their choice of medical treatment behaviour and indirectly show the underlying social differentiation.

### 4.4. Application to Public Health Policy

Recently, China’s urbanization construction has entered a new stage. According to the resident population and service radius, guaranteeing the rational distribution of medical and health facilities and the realization of fair and equal basic medical resources is the core concept of China’s Healthy City Strategy [42,43,44]. At the same time, China will also improve the medical service system, promote the equalization of public health resources, provide innovative medical service supply models, improve the quality of medical services, and satisfy the demand for medical treatment for patients with chronic diseases to achieve the core aims of the systematic construction of medical and health services. China is becoming an ageing society. The proportion of chronic diseases, mainly cardiovascular and cerebrovascular diseases, diabetes and respiratory diseases, is rising rapidly among older persons. This situation requires community hospitals to meet the daily medical needs of local residents in a convenient manner. In addition, the community medical system vigorously promoted by the state has also promoted the care for and prevention of chronic diseases as important aspects of the construction of a healthy living community [45,46,47,48].

## 5. Conclusions

### 5.1. Key Findings

This paper takes Gaoyou City as a case study and analyses the balance of medical supply and demand at the block scale by investigating the characteristics of patients’ medical choices. In neighbourhoods with medical supply-demand imbalance, the factors influencing patients’ medical choices are analysed considering built environment factors, subjective evaluation of medical institutions, and individual socioeconomic background. There are significant differences in the choice of medical institutions for patients with chronic diseases with different socioeconomic backgrounds, medical insurance options, and living conditions. In addition, the research has further expanded the existing theoretical model of medical needs and incorporated the environmental factors of residential areas into the medical treatment choice index system. The cross-influences of economic level, self-rated disease severity and evaluation of low-income medical institutions were added to the model to more effectively measure the impact on patients’ medical choices.

At present, China’s basic medical insurance system has problems, such as a low compensation rate, a limited scope of diagnosis, treatment and drug reimbursement, and inadequate funding and facilities for primary medical institutions. Among the respondents who participated in the survey, 73.5% indicated that most of their chronic disease expenses were difficult to reimburse, which indirectly leads patients to choose to self-medicate, such as by buying medicines nearby. Older people with insurance generally seek high-level medical institutions after they become ill. Insufficient qualifications and service conditions in primary medical institutions are the main reasons for patients choosing a high-level institution. The young people are more likely to go to community hospitals for medical treatment. This phenomenon may be related to the young group’s education level, health awareness and living habits. At the same time, it may also be affected by China’s current medical policy, family member structure, economic level and other factors. The large medical institutions in the city diagnose and treat patients with acute illness, while the community medical institutions around the residential areas pay more attention to medical treatment needs, such as daily medical examinations and reviews. When undertaking the construction of medical and health services in the next stage, it is necessary to pay full attention to the convenience and popularization of community medical institutions in the prevention and treatment of chronic diseases, actively redistribute the pressure of diagnosis and treatment experienced by large medical institutions, and meet the needs of patients with chronic diseases.

### 5.2. Implications

The level of supply and demand for medical services are closely related to factors such as the socioeconomic attributes of patients, disease type, medical policies, and levels of urban spatial development. Subsequent research will further analyse the neighbourhoods where patients’ medical space is poorly configured. Based on the patients’ socioeconomic background factors, the analysis will include the influence of government policies, family background, economic situation, willingness to visit, health cognition, self-diagnosis and other factors on patients’ medical behaviour. Although there are reports in the literature that there is a connection between residents’ living habits and medical choices, there is a lack of in-depth analysis of the specific influencing factors and mechanisms of action [49,50]. Follow-up studies will focus on the impact process of patients’ daily living habits, pedestrian neighbourhood usage, fitness space participation and other activities. Further research will provide a decision-making reference for the refined layout and functional improvement of improved urban medical facilities and will provide useful suggestions for patients’ choice of medical institutions and the cultivation of daily healthy living habits [51,52].

At present, few studies focus on the relationship between living habits and medical choices in China. In the next stage, we will delve into the relationship among daily living habits, eating habits, exercise habits and other lifestyle habits on medical choices. This paper analysed the accessibility of medical services only from the geospatial level, but lacks a comprehensive analysis of the accessibility of medical technology and service capabilities. Meanwhile, we analysed the public transportation factors in the traffic environment, but lacks comprehensive analysis of other traffic conditions. In the subsequent research, these contents will be analysed in depth. In addition, this paper lacks a comparative study on the behaviour of patients with chronic diseases and other types of diseases. Because the research on patients’ medical behaviour has just begun in China, few studies focus on the relationship between patients’ psychological perception and medical choice. Therefore, in the next stage, we need to learn more from the existing research results and build a research system and policy plan applicable to China’s national conditions.

### 5.3. Limitations and Future Research Directions

In this paper, there have limitations in the setting of the research unit. We selected a block scale as a research unit. However, it is possible that the patient’s daily medical treatment is not limited to the local block, and it may lead to the occurrence of Uncertain Geographic Context Problem (UGCoP) and causes correlation analysis deviations between factors in mechanism analysis The subsequent research can further investigate the actual activity trajectory and activity boundary of the patients accurately through more in-depth interview research or using GPS trajectory recording equipment. Therefore, it can identify the patient’s living space boundary and the organization of built environment elements more accurately, and provide more detailed data support for in-depth study of the mechanism of different types of built environmental factors affecting patients’ medical choice. It will provide more detailed data support for in-depth study of the mechanisms of different types of built environmental factors affecting the patients’ medical choice. The medical choice of patients with chronic diseases is a multi-factor complex process, which influenced by many factors, such as the patient’s own health perception, medical service supply and built environment factors. The sample size, research period, and the local social and cultural environment also have an impact on the patients’ medical choices. Therefore, the application of the research results in this paper will be limited by regional and time constraints. Although this article considers many factors that influence the choice of medical treatment, we didn’t deeply analyse the interaction between different factors. It is impossible to clarify the importance of different influencing factors by using only logistic regression models. Therefore, the public health policy recommendations given in this study are not specific enough. Functional ability is the actual or potential capacity of an individual to perform the activities and tasks that can be normally expected. A given function integrates biological, psychological and social domains. But we didn’t include functional ability as a variable in this study. It should be noted that this article is the initial work of our research, and we will continue to include more influential factors in later research.

## Figures and Tables

**Table 1:** Basic information of interviewees (*n* = 1139).

Item	Group	Proportion (%)	Item	Group	Proportion (%)
Age	Less than 31 years old	3.9	Monthly household income	Below 3000	28.6
	31–45 years old	17.4		3000-5000	36.1
	45–60 years old	37.2		5000-8000	27.1
	above 60 years old	41.5		8000 and above	8.2
Gender	Male	62.5	Medical insurance	No insurance	52.5
	Female	37.5		Social medical insurance	37.2
Education level	Primary school and below	37.2		Commercial medical insurance	10.3
	Junior high school	44.6	Family Size	Two or below	17.6
	High school	16.5		Three	33.5
	Bachelor degree and above	1.7		Four	43.8
Marital status	unmarried	37.2		Four and above	5.1
	married	43.5	Self-rated disease severity	Light	33.6
	Inconvenient to answer	19.3		General	21.7
Disease type	diabetes	48.7		Serious	30.1
	hypertension	27.9		Inconvenient to answer	14.6
	hyperlipidemia	38.9			
	Other chronic diseases	54.5			

**Table 2:** The conditions of medical institutions and other built environment elements.

Item	Group (Proportion)	Description
**Medical institutions (*n* = 45)**		
Type	Village clinics (23.7%), township hospitals (27.1%), private clinics (12.4%), county-level and above medical institutions (36.6%)	Chinese medical institutions are divided into different levels based on their service population and radius.
**Subjective evaluation of medical institutions (*n* = 1139)**		
Medical fee satisfaction	Satisfaction (47.2%); Dissatisfaction (52.8%)	A questionnaire form was used to obtain the subjective evaluation results of patients, which were divided into two categories: Satisfaction and dissatisfaction.
Medical service satisfaction	Satisfaction (53.1%); Dissatisfaction (46.9%)	
Medical environment satisfaction	Satisfaction (39.7); Dissatisfaction (60.3)	
**Other built environment elements (*n* = 1139)**		
Public transportation convenience	Within 5 min (32.5%); 5–10 min (27.4%); 10–15 min (23.8%); more than 15 min (16.3%) (for public transport users)	We obtained the traffic environment around the patient’s residential area through questionnaires. Walking time from the patient’s place of residence to the common public transportation station.
Medical accessibility	Within 5 min (27.3%); 5–10 min (29.1%); 10–15 min (36.2%); more than 15 min (7.4%)	Public travel time required for patient’s usual medical treatment behaviour.

**Table 3:** Comparison of the goodness of fit among the three models.

Model Type	Estimated Parameter Number	Log-Likelihood	AIC	BIC
Multiple logit model	7	−117.418	944.837	782.962
Conditional multiple logit model	12	−110.635	825.269	766.798
Nested multiple logit model	12	−35.482	645.965	483.434

AIC: Akaike information criterion, BIC: Bayesian information criterion.

**Table 4:** Basic information on medical institutions at different levels

Type	Grade	Service Radius (Meter)	Number	Number of Serviced Blocks
County-level and above hospital	3	2000 m	4	12
Township-level hospital	2	1500 m	17	18
Village-level clinic	1	800 m	23	32

**Table 5:** Nine classifications for the medical supply and demand balance at the block scale.

Supply and Demand Balance Index		Local Medical Demand		
		Low Level	Medium Level	High Level
Local medical supply	Low level	Low level balance	Low supply—medium demand	Low supply—high demand
	Medium level	Medium supply—low demand	Medium level balance	Medium supply—high demand
	High level	High supply—low demand	High supply—medium demand	High level balance

**Table 6:** Nested multiple logit model results of the chronic disease patients’ chosen institutions (control group: Village clinic).

Explanatory Variables	Township-Level Hospital		Private Clinic		County-Level and Above Hospital	
	RC	SD	RC	SD	RC	SD
**Built environment features**						
Public transportation convenience (walking time to surrounding bus stops)						
5–10 min	−0.072	0.142	0.104	0.274	0.135	0.255
10–15 min	0.036	0.085	−0.096	0.158	0.094 *	0.176
More than 15 min	0.067 *	0.153	−0.081 *	0.155	0.046 **	0.113
Medical accessibility (average travel time for daily medical treatment)						
5–10 min	0.082	0.135	0.014 *	0.035	0.023 **	0.132
10–15 min	0.014	0.025	−0.039	0.122	0.032 *	0.113
More than 15 min	−0.025	0.032	−0.048 **	0.103	0.015 *	0.096
**Socioeconomic factors**						
Age						
31–45	0.036 **	0.151	0.031	0.136	−0.043	0.056
45–60	0.076	0.132	−0.057 *	0.194	0.132 **	0.226
Above 60	0.107 **	0.256	−0.135	0.226	0.105	0.172
Sex (female)	0.024	0.035	0.056	0.133	0.079 **	0.145
Educational level						
Primary school	−0.056	0.124	0.034 **	0.045	−0.062	0.131
Junior high school	−0.037	0.102	0.068 *	0.125	0.076	0.158
High school and above	0.073	0.145	−0.024	0.043	0.055 ***	0.142
Marital status (married)	0.027	0.035	0.022	0.046	0.013	0.037
Average monthly household income						
3000–5000	0.076	0.134	0.018 **	0.033	0.041	0.107
5000–8000	0.052 *	0.182	−0.034	0.107	0.046 **	0.072
Above 8000	0.041	0.152	−0.033	0.152	0.105 ***	0.234
Medical insurance						
No insurance	0.074	0.172	0.057 **	0.085	0.105	0.093
Social medical insurance	0.093 *	0.131	0.047	0.092	0.133 **	0.243
Commercial medical insurance	0.062	0.152	0.042	0.093	0.072 *	0.114
The length of time since diagnosis						
Half a year to one year	0.073 *	0.131	0.047	0.092	0.133 **	0.243
One year to two years	0.062	0.152	0.042 *	0.093	0.072 *	0.114
More than two years	0.041	0.152	−0.033 **	0.152	0.105	0.234
Self–rated disease severity						
Light	0.046 *	0.175	0.034 *	0.045	−0.046	0.142
General	0.037	0.092	0.092	0.187	0.029 **	0.069
Serious	0.066 *	0.139	0.086	0.137	0.035 ***	0.122
**Subjective evaluation of medical institutions (not satisfied)**						
Satisfied with medical fee	0.236	0.324	−0.087	0.154	0.042 **	0.127
Satisfied with medical service	0.313 ***	0.646	0.114 *	0.223	0.031	0.095
Satisfied with medical environment	0.082 *	0.148	0.124	0.277	0.065 **	0.152
**Multi–factor crossover**						
Prevalence level*poverty						
Light*poverty	0.046 *	0.125	0.082	0.137	0.127 ***	0.253
General*poverty	0.053	0.135	0.018 ***	0.097	0.142	0.228
Serious*poverty	0.104	0.177	0.052 **	0.129	0.122 *	0.282
Medical charge*poverty	0.131	0.218	−0.088	0.159	0.095 **	0.182
Medical service*poverty	0.087	0.205	0.116 **	0.249	0.083	0.276
Medical environment*poverty	0.122	0.298	−0.153	0.228	0.117	0.283
Number of samples	331		483		325	
Log likelihood value	−308.736					
Wald chi^2^ (92)	77.39					
Significant level	0.000					
LR test for IIA						
Chi^2^ (2)	23.15					
Prob > chi^2^	0.0000					

Notes: ***, **, and * represent variables at significance levels of 1%, 5%, and 10%, respectively; RC refers to regression coefficient; SD refers to standard deviation.

## Appendix A. Questionnaire about medical choice for chronic disease

To whom it may concern,

This is an academic questionnaire about medical choice for the disease. Because of your participation that determines the success of this study, so I hope you can participate in this survey. Please fill in this questionnaire in according to actual condition, and we confirm that the results will be only used for academic statistical research rather than other use in any form. Your participation is anonymous. If you have any discomfort during the questionnaire process, you can interrupt it at any time. We promise not to report or disclose your personal information in any way.

Thanks again for your kind help to fill in the questionnaire.

**Section I Your basic information**
1. What is your sex? □Male □Female 2. What is your age? □Less than 31yr □31–45yr □45–60yr □above 60yr 3. What is your education level? □Primary school and below □Junior high school □High school □Bachelor degree and above 4. How many people are there in your family? □Two or below □Three □Four □Four and above 5. How much is your household income of one month (RMB)? □Below 3000 □3000–5000 □5000–8000 □8000 and above 6. What is your marital status? □unmarried □married □Inconvenient to answer 7. What is your medical insurance? □No insurance □Social medical insurance □Commercial medical insurance 8. Would you willing to participate in our follow-up research work? □Yes □No ---------------------------------------------------------------------------------------------------------------------------
**Section II Your physical health status**
9. How do you feel about your physical health status? □Very well □Good □Not well □Inconvenient to answer 10. Height: _________ meters; weight: _________ kg 11. Do you have diabetes: □Yes □No; And the length of time since diagnosis_________ 12. Do you have hypertension: □Yes □No; And the length of time since diagnosis_________ 13. Do you have hyperlipidemia: □Yes □No; And the length of time since diagnosis_________ 14. Do you have other chronic diseases? (Such as coronary heart disease, arthritis, stroke, etc.) □Yes □No; If you have, the disease is_________; and the length of time since diagnosis_________ 15. How do you assess the degree of your illness? □Light □General □General □Inconvenient to answer ---------------------------------------------------------------------------------------------------------------------------
**Section III Your medical choice behavior**
16. Have you had a corresponding medical treatment for your own chronic disease in the past six months? □Yes □No; 17. Do you choose fixed hospital for your medical treatment? □Yes □No; 18. The institution’s name for your medical treatment? _________ 19. What kind of traffic travel mode you usually choose for your medical treatment? □walk □ride □bus □self-driving 20. If you use bus travel mode, how long did it take from your residence to the common bus stop by walking? □Within 5 min □5–10 min □10–15 min □More than 15 min 21. How long did it take from your residence to the hospital by bus? □Within 5 min □5–10 min □10–15 min □More than 15 min 22. Are you satisfied with the hospital’s medical charge level? □Satisfied □Not satisfied 23. Are you satisfied with the hospital’s medical service level? □Satisfied □Not satisfied 24. Are you satisfied with the hospital’s medical environment level? □Satisfied □Not satisfied
